# Computational Tension Mapping of Adherent Cells Based on Actin Imaging

**DOI:** 10.1371/journal.pone.0146863

**Published:** 2016-01-26

**Authors:** Ian Manifacier, Jean-Louis Milan, Charlotte Jeanneau, Fanny Chmilewsky, Patrick Chabrand, Imad About

**Affiliations:** 1 Aix-Marseille Université, ISM, CNRS, UMR 7287, Marseille, France; 2 APHM, Institute for Locomotion, Sainte-Marguerite Hospital, 13009, Marseille, France; Institut Albert Bonniot-INSERMU823, FRANCE

## Abstract

Forces transiting through the cytoskeleton are known to play a role in adherent cell activity. Up to now few approaches haves been able to determine theses intracellular forces. We thus developed a computational mechanical model based on a reconstruction of the cytoskeleton of an adherent cell from fluorescence staining of the actin network and focal adhesions (FA). Our custom made algorithm converted the 2D image of an actin network into a map of contractile interactions inside a 2D node grid, each node representing a group of pixels. We assumed that actin filaments observed under fluorescence microscopy, appear brighter when thicker, we thus presumed that nodes corresponding to pixels with higher actin density were linked by stiffer interactions. This enabled us to create a system of heterogeneous interactions which represent the spatial organization of the contractile actin network. The contractility of this interaction system was then adapted to match the level of force the cell truly exerted on focal adhesions; forces on focal adhesions were estimated from their vinculin expressed size. This enabled the model to compute consistent mechanical forces transiting throughout the cell. After computation, we applied a graphical approach on the original actin image, which enabled us to calculate tension forces throughout the cell, or in a particular region or even in single stress fibers. It also enabled us to study different scenarios which may indicate the mechanical role of other cytoskeletal components such as microtubules. For instance, our results stated that the ratio between intra and extra cellular compression is inversely proportional to intracellular tension.

## Introduction

The cytoskeleton of human adherent cells is capable of generating forces, which enables a cell to pull on its surrounding environment, spread and move [1][2]. On the other hand, cells are not simply capable of generating forces, they are sensitive to mechanical stimuli as well[1][2]. Cells are so sensitive to mechanical cues that it may influence survival or death, protein synthesis, cell division, migration or differentiation [2][3][4]. The phenomenon by which mechanical stimuli influence living cells is known as mechanotransduction. Decades of research in this field has provided some details on how mechanotransduction works. On the other hand, the combined mechanical interactions of the main cytoskeletal components such as FA, actin [5][6][7], microtubules [7], intermediate filaments and the nucleus, are still miss understood. For instance, some experimental results clearly indicate that actin and microtubules behave as distinct mechanical structures, where actin filaments exert tension and microtubules bear compression forces [8][9]. The cytoskeleton can therefore be seen as a structure of tensegrity, in which tension generated by actin stress fibers can be viewed as the initial and predominant force which mechanically loads the structure: in vitro experiments indicated that actin depolymerization using drugs can decrease cellular stiffness by 85% [9]. Cross-link proteins reticulate the filamentous network, which leads to greater structural stiffness. For instance, alpha-actinin bundles link actin filaments together so as to form large stress fibers. On the other hand the generation of tension force transiting within an actin fiber either requires stretching or the involvement of motor proteins, such as myosin. For instance, one myosin protein (myosin II), links two actin filaments together and generates contraction force by displacing filaments relatively to one another when supplied with ATP. According to experimental findings [10], in static non deformed adherent cell, this phenomenon generated a contraction of the actin stress fibers equivalent to a stretching of about 20%.

Microtubules, which are described as compression bearing elements, contributing to the overall structural rigidity and shape of the cell, are large polymeric tubes generally emitted from the centrosome. The experimental observation of in vitro cells show that cellspull slightly more on deformable substrate after microtubule depolymerization, this clearly indicates that microtubules withstand intracellular tension generated by the actin network[11]. However several studies tend to diminish their mechanical role; for instance, cellular rigidity is only slightly influenced by microtubule depolymerization [9]. Furthermore, it has been shown that the ratio of compression forces supported by microtubules is inversely proportional to cell spreading and that intracellular tension in highly spread cells is largely balanced by the extracellular matrix (ECM) [11]. Cytoplasm, microtubules and ECM are thus complementary load bearing elements, even if most of the compression load is supported by the ECM.

The cell is composed of different types of filaments (actin, intermediate filaments, microtubules…) with specific structural arrangements. Most analytical methods are unable to grasp the complexity of such filamentous systems. To map internal forces transiting through each of these networks, development of computational cell models is needed. Indeed such tools could have many advantages, they could help researchers to see and quantify the influence of various biological and mechanical parameters. Many computer models were previously created in order to determine the mechanical state of adherent cells [12][13][14]. In line with classical tensegrity models, our group has developed a computational approach based on divided medium mechanics. It represented the cytoskeleton composed of a high number of filaments by an equivalent multi-interaction system [12][15]. However, these previous models could not mimic the real spatial distribution of the cytoskeleton. Very recently a model developed by Soine et al. did, use stress fiber imaging to generate a stress fibers like finite element model that generated equivalent tension to that of their real counterpart. Even if Soine et al.’s approach was a significant advancement to the development of a biofidelic cell model, it still lacks some crucial advantages. Firstly, Soine et al.’s approach necessitated highly visible and distinctive stress fibers, limiting the models application to specific cell types. Secondly, Soine et al.’s model only represents tensile elements capable of representing mechanical forces and optimizes the amount of force applied on each element, the problem being that this system has a high number of degrees of freedom[16].

We therefore focused our work on the development of a new modeling approach which also represents the structural specificities of the true cellular structure. The model was generated from focal adhesion, the nucleus and the actin network imaging of an adherent cell. This approach has the advantage of being cell-dependent: the model numerically reproduces the pre-stressed actin cytoskeleton of any adherent cell, and enables quantification of local intracellular forces. We assumed that high tension forces transit through large actin stress fibers and that regions of high actin density revealed by immunostaining are regions of high tension. So pixels from the actin image were transformed into mechanical nodes to generate a system of contractile interactions representing the cytoskeleton. Each node was assigned a label in correspondence to the gray value of the paired pixels. Interactions were set to be in accord with the spatial distribution of actin filaments as can be observed on the picture: nodes corresponding to pixels with high actin density interacted between each other via high tension forces, while nodes with labels corresponding to low amounts of actin would interact with weaker interactions. At this stage, the global level of tension still had to be identified. To do so, we used experimental forces exerted by the cell on FA and compared the sum of its magnitudes, to the sum of focal adhesion force magnitudes (FA forces) generated by the cell model. All stiffness values of contractile interactions composing the model were adapted in proportion to actin concentration so as to obtain a contractile system capable of generating FA forces similar to the ones measured experimentally. Based on previously published studies, we considered FA force values to be proportional to experimentally observed FA size.

In addition, based on the fact that intracellular forces are at equilibrium, we incorporated a compressive interaction law between nodes so as to balance intra-cellular tensile forces. This law generated repelling compression forces preventing nodes from collapsing on one another. These forces then formed a network which mechanically represented compression bearing cytoskeletal elements such as microtubules.

Our innovative modeling approach was here applied on a collection of 30 adherent cells to reconstruct in every cell the actin cytoskeleton and precisely determine the local stiffness as well as the distribution of tensile forces. After computation, the model allows estimation of tension within a stress fiber in every part of the cell, directly from the image of its actin network.

## Materials and Methods

To compute intracellular forces in an adherent cell we generated a mechanical model based on fluorescent images of the actin network, focal adhesions and the nucleus. We applied the model on 8 cells from a population of dental pulp fibroblasts cultured in adhesion on a flat glass substrate. Pixels representing the stained cell were converted into mechanical nodes. A label was attributed to each node depending on what it represented: actin and its level of expression, vinculin or nucleus. This method allowed us to generate a mechanical interaction between each node and its neighbors according to their respective labels and in order to accurately represent local mechanical properties. On the one hand, vinculin labelled nodes were immobilized so as to act as the mechanical anchor points between the cell and the substrate. On the other hand, actin labelled nodes were connected to each other via neighbor to neighbor tensile interactions which formed a contractile network representing the actin network of the cell.

### Biological protocol

#### Culture of human pulp fibroblasts

Human pulp fibroblasts were prepared from immature third molars. Briefly, the teeth were obtained from subjects between the age of 16–21 years old in compliance with French legislation.

Since wisdom teeth extracts are medical wastes, an Ethics Committee approval was not necessary. No Ethics Committee was consulted for this work.

The wisdom teeth were obtained by Dr. Jean-Charles Gardon from subjects between the age of 16–21 years old in compliance with French legislation. Dr. Jean-Charles Gardon (the oral surgeon) was responsible for acquiring written informed patient consent and if needed of the legal guardians. Extracted teeth were then given to Ph. D. Charlotte Jeanneau by Dr. Jean-Charles Gardon for pulp extraction, no patient specific information was given neither to her nor to any of the authors.

To ensure full anonymity teeth sample extraction dates were not recorded. The authors did not have access to any identifying patient information. As required by French law, patient identification is impossible based on records, even by combining Dr. Jean-Charles Gardon's and the authors' records.

After extraction, the teeth were washed and the apical portions removed. The extirpated dental pulp was minced, and explants were cultured in 100-mm-diameter culture dishes containing minimum essential medium (MEM) supplemented with 10% fetal bovine serum, 100 UI mL−1 penicillin, 100 μg mL−1 streptomycin and 0.25 μg mL−1 amphotericin B and placed at 37°C into a humid incubator with an atmosphere of 5% CO2. Cells from confluent cultures were collected by trypsinization and subcultured in T75 flasks.

#### Cell imaging by immunofluorescence double-staining

Pulp fibroblasts cells were allowed to spread for a period of time of 24h in 8-well glass culture chambers. Isolated cells were fixed with 4% paraformaldehyde for 20 min at 37°C. After washing with phosphate buffered saline (PBS), cells in the chambers were incubated in blocking/permeabilization buffer (3% BSA, 0.3% Triton X100 in PBS) for 45min at room temperature an incubated overnight at 4°C with mouse anti-human vinculin IgG (1/100), diluted in PBS containing 1% BSA and 0.3% TritonX100. After washing with PBS 0.1% bovine serum albumin (BSA), cells were incubated for 45 min with the fluorescent secondary antibodies Alexa Fluor-594 goat anti-mouse IgG (1/400), phalloidin conjugated with Alexa Fluor-488 (1/2000) for actin staining, and DAPI (1μg/ml) for nuclear counterstaining, all diluted in PBS containing 1% BSA and 0.3% TritonX100. After washing with PBS, the slides were mounted with Glycergel, kept at 4°C and visualized with fluorescence equipped light microscope (Axio Observer.A1, Carl Zeiss Microscopy, Jena, Germany). Phalloidin stained actin, vinculin, and DAPI were respectively captured on three different color channels (RGB) so as to observe each cellular component separately. 3 images of same size and resolution were obtained, one representing actin, another vinculin and the last one the nucleus. Each image was treated separately. The image representing vinculin was treated to increase contrast on focal adhesions. Once extracted from the image, the cell is 62 μm in length, 35 μm across and is defined by about 35 000 pixels. Images were stored using the TIFF format to avoid information loss.

#### Estimating focal adhesion forces

A linear relationship has been reported between the area covered by vinculin in focal adhesions and the value of traction forces magnitudes transiting through focal adhesions [17]. The expressed relationship is 5.5 nN/μm^2^. Other studies confirmed the existence of a linear relationship between focal adhesion size and force intensity [18][19]. On the other hand, one of these studies argued that this relationship only applies to focal adhesions larger than 1 micrometer squared [18]. Thus, in this study, we only considered focal adhesions larger than 1 μm^2^.

### Constitutive equations of the cell model

#### Tensile interactions to represent actin filaments

To numerically recompose the actin network, pixels were converted into actin nodes. According to computational limitations, the number of nodes was set to remain below 10 000. This led to the definition of one node per group of 4 connected pixels; distance between neighboring nodes was 0.58 μm. Actin nodes were then connected to each other by tensile elastic interactions which generated a pre-stressed network, so as to simulate the contractile nature of the actin network. These tensile interactions (or inter-tensions) followed a law which was described in our group’s previous studies [12][15]. They behaved as virtual pre-strained elastic rubber bands between all neighboring actin nodes of the model. Furthermore, those interactions would generate a traction force which was proportional to stretch (strain), or became null when the virtual rubber band slackened. As shown in the set of Eq 1, the traction force ***T*** was function to the value gap ***g*** (the distance between two nodes), ***g***_***0***_ being the gap at the beginning of the simulation, the stiffness ***K*** > 0, ***ε***_**0**_ > 0 the pre-strain in the virtual elastic rubber band and **g**_**v**_ the maximal gap beyond which the interaction would not be created.

$$\begin{array}{cc} \{\begin{array}{c} g\in \;]\left(1-\varepsilon_{0}\right)g_{0};g_{v}\;]\Rightarrow T=-K\left(\frac{g-g_{0}}{g_{0}}+\varepsilon_{0}\right)\\ g\in \left[0;\left(1-\varepsilon_{0}\right)g_{0}\;\right]\text{or}\;g\geq g_{v}\Rightarrow T=0 \end{array} & \end{array}$$ (1)

The stiffness ***K*** of the elastic rubber band like interaction was defined as a force per strain. Defining a positive constant pre-strain value ***ε***_**0**_ generated at the beginning of the simulation non-zero inter-tensions between nodes and so reproduced the contractile nature of the cell. Deguchi et al. experimentally found that actin stress fibers are pre-strained by about 20% [10]. We thus applied 20% of pre-strain *ε*_0_ to all actin tensile interactions in order to generate initial contractile inter-tensions at null deformations. On the other hand, to limit the number of tensile interactions, the tensile interaction law was given a visibility threshold gap *g*_*v*_ greater than *g*_0_, the initial distance between two closest actin bodies, and lower than $\sqrt{2}g_{0}$ so that diagonally disposed neighboring bodies would not be able to interact. We thus arbitrarily set *g*_*v*_ equal to 1.1*g*_0_.

All these tensile interactions between nodes formed a pre-strained tensile network of interconnected nodes. During computation, all actin and nucleus nodes, were free to move until the whole tensile network reached a mechanical equilibrium, which consequently led to the slight adjustment of the magnitude of local tensile forces.

#### Modelling Nucleus and Focal Adhesions

Nucleus and vinculin gray scaled images were binarized, which enabled the respective conversion of corresponding pixels into nucleus and vinculin nodes. Groups of connected pixels were automatically identified from the vinculin image as focal adhesions using the ImageJ software [20][21]. Their areas were reported in this study so as to estimate the magnitudes of traction forces transiting through them.

The same interaction laws and parameters as in actin tensile interactions were used to generate actin-vinculin interaction and actin-nucleus interactions as well. Except that nucleus interactions were given a homogenous stiffness 10 times greater than the strongest actin interaction.

#### Modeling microtubules

Microtubules are known to bear intra-cellular compression forces. Since we lacked experimental data to represent microtubules based on direct experimental observation, we thus decided to generate a coherent intracellular compression network between the nodes of the model by individually surrounding them with small rigid impenetrable spherical contactors. When brought in contact with one another, the spherical contactors would interact by generating repulsive forces. This method thus generated an intracellular network of compression forces opposed to the actin tension network. To achieve this compression bearing function, we coupled sphere contactors to a frictionless contact law as follows, where ***g*** is the gap between the contactors and ***R***_***N***_ the normal reaction force.

$$\begin{array}{cc} g\geq 0\;;R_{N}\geq 0\;;g\cdot R_{N}=0 & \end{array}$$ (2)

### Parametric analysis to determine intracellular forces

#### Linking tensile interaction stiffness with local actin density

The stiffness values of all actin inter-tensions were set to be proportional to local actin density expressed by actin image gray values. This method allowed us to take into account the heterogeneous distribution of an actin filament in the model and its effect on cellular mechanics. Gray values of actin image, noted ***c***, were first normalized, such that *c* ∈ ]0; 1]. The value 0 represents a dark area, with no actin; while the value 1 represents a white area, with the highest amount of actin. Pixels from actin images were categorized in 10 different labels ranging from ***l***_***1***_ to ***l***_***10***_ corresponding to 10 shades of gray. With ***l***_***1***_ being the label representing the lowest actin density, while ***l***_***10***_ was the label corresponds to the highest actin density, such that:

$$\begin{array}{cc} \forall c\in ]0;1]\Rightarrow \exists !i\in 1;10\;such\;that\;c\in C_{i}=]0.1\cdot \left(i-1\right)\;;0.1\cdot i]\Rightarrow l_{i} & \end{array}$$ (3)

Labelled pixels were then converted into labelled actin nodes. 10 elastic laws (Eq 1) were created to rule interactions between nodes of a same label. The ***K***_***i***_ stiffness value of each of these 10 interaction laws was defined to be proportionally to the highest gray value of its respective *l*_*i*_ value. For instance, for a given label valued ***i***, the stiffness ***K***_***i***_ is given by the following equation:

$$\begin{array}{cc} K_{i}=a*c_{i\;max} & \end{array}$$ (4)

where c_(i max)_ = 0.1∙i, is the highest gray value contained within ***C***_***i***_.

Interactions between nodes with two different labels interacted as if they were both from the weakest label. The method we developed to calculate the value of the coefficient ***a***, linking stiffness to actin density, will be the topic of the next sub-section.

#### Solving the equation between model stiffness and actin density

The following solving method was used on each of the 8 cells individually. Solving Eq 4, means finding the coefficient **a**. Tensile interactions were pre-stressed and would thus spontaneously generate tension forces, which consequently made the cell model pull on its focal adhesions. To solve for ***a***, we considered the fact that increasing ***a*** would increase the sum of pulling force on focal adhesions, or pull less if ***a*** decreased. We here applied an inverse mechanics approach to determine the structural properties of the cell model at hand based on its mechanical response. We thus tested different values of ***a*** and then compared the total of force magnitudes applied on focal adhesions computed by the model to the ones estimated experimentally. Based on the results we obtained, we were able to linearly interpolate the best value of ***a***. Once we found the correct value of ***a***, we considered our model to be an optimal match to the true actin contractile structure of the cell.

The adherent cells we observed were fixed and thus supposed to be at a mechanical equilibrium. To be consistent, the multi interactions system which constituted the model had to converge to a mechanical equilibrium as well. Computations were led using the LMGC90 solver, dedicated to divided medium mechanics and multi interaction systems. For every value of **a**, the mechanical behavior of the model was computed over a period of 300μs divided in 600 steps.

Nodes were given a mass equivalent to a volume of water enclosed by the contactor envelopes. Node masses were all equal to 0.8pg.

During computations, the model could rearrange. We nonetheless hypothesized that tensile interaction strain would not vary significantly along the stress fibers. This means that tension forces would remain the same along stress fibers throughout the computation, and that the force between two nodes of the label ***i*** was only due to the stiffness ***K***_***i***_ and the pre-strain ε_0_ = 0.2.As ***K***_***i***_ depends on ***c***_***i max***_ (the corresponding gray value), we can argue that interaction forces ***T*** located between two nodes depends on ***c***_***i max***_ as expressed in the following equation:

$$g\approx g_{0}\Rightarrow T\approx -K_{i}\;.\varepsilon_{0}\approx -a*c_{i\;max}\;.\varepsilon_{0}$$ (5)

After the determination of the value of the parameter ***a***, the pre-stressed contractile cell model we obtained was able to give the values of intracellular tensions. We then introduced ***T***_***mean***_ as the mean value of all inter-tensions outputted by the model.

#### Cross-linear tension forces within the modeled cell

After setting ***a***, the number of interaction and the force value per interaction given by the model still depended on its spatial resolution. The resolution of the model was determined by the minimum distance between two nodes which delimited the region modeled by each interaction. Indeed a low resolution model will be modeled by a proportionally low number of interactions, thus implying high force magnitudes per interaction, while a higher resolution model would be represented by a higher number of interactions, leading to low force magnitudes per interaction. On the other hand, the cross linear density of force remains the same in both cases, since it is resolution independent. Thus, in order to estimate the intra-cellular density of tension within the model, while remaining independent to the resolution of the model i.e. the mesh size, we considered ***T***_***cl***_ the cross-linear tension (or cross-linear density of tension) defined as:

$$\begin{array}{cc} T_{cl}=\frac{T}{d_{0}}\approx -a*c_{i\;max}\;.\frac{\varepsilon_{0}}{d_{0}} & \end{array}$$ (6)

As a consequence we could define ***T***_***cl-max***_ as the cross linear density of force corresponding to the highest value of actin density, i.e. which relates to the highest actin concentration represented by label number 10 (***c***_***10 max***_ = 1).

$$\begin{array}{cc} T_{cl-max}\approx -a*c_{10\;max}\;.\frac{\varepsilon_{0}}{d_{0}}=-a\;.\frac{\varepsilon_{0}}{d_{0}} & \end{array}$$ (7)

We also introduced ***T***_***mean-cl***_ the cross linear mean inter-tension defined as:

$$T_{mean-cl}=\frac{T_{mean}}{d_{0}}$$

with *T*_*mean*_ being the mean tension value of all actin interactions.

#### Calculating tension within an actin stress fiber in the imaged cell

The model allowed us to determine the density of tensile force in the model ***(T***_***cl***_***)*** and its highest value ***(T***_***cl-max***_***)*** corresponding to the highest actin density. To estimate tension transiting through an actin stress fiber inside the cell, we measured the fiber diameter ***(∅***_***SF***_***)*** and the mean gray value ***(c***_***mean***_***∈[0;1])*** using a custom written Matlab^TM^ script. Which then enabled us to calculate the amount of tension transiting through the given stress fiber ***(T***_***SF***_***)*** with the following equations:

$$\begin{array}{cc} T_{SF}=T_{cl-max}\cdot {\bar{c}}_{cross}\cdot \emptyset_{SF} & \end{array}$$ (8)

## Results

### From focal adhesion size to cytoskeleton pulling force

**Fig 1:**
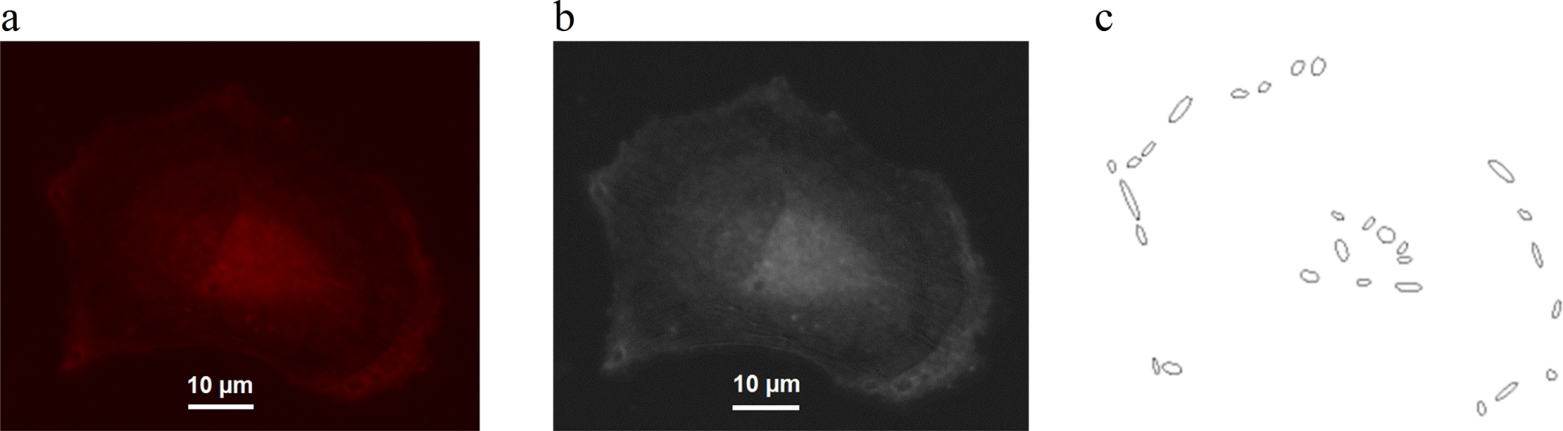
Focal adhesions. Cell Example: a) vinculin staining b) vinculin staining & view of focal adhesions c) focal adhesions larger than 1 μm^2^.

**Fig 2:**
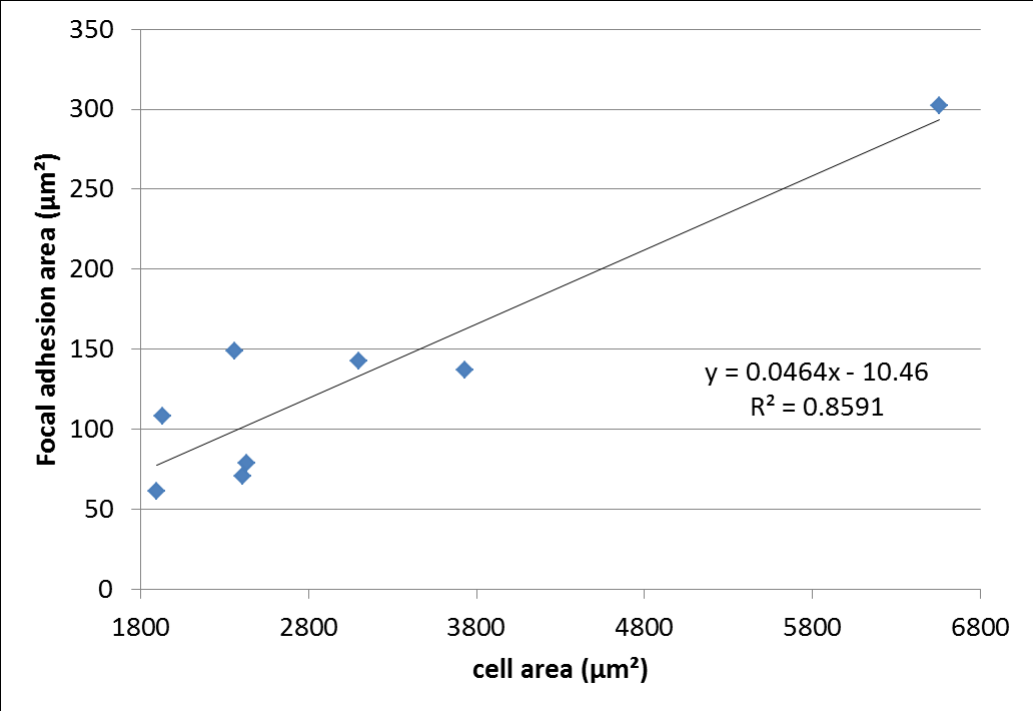
Focal adhesions area vs cell area. We notice a linear relationship between focal adhesion area (μm^2^) and cell size (μm^2^) for 8 cells.

Pulling force magnitudes exerted on focal adhesions by the cytoskeleton were determined in correlation with their size, based on vinculin fluorescence imaging (Fig 1). In this study we only considered focal adhesions larger than one squared micrometer (Fig 2). Sub micrometer focal adhesions were not taken into account because there is no correlation between their size and the magnitude of the pulling force which transit through them.

### From cell imaging to a biofidelic reconstruction of the contractile actin network

**Fig 3:**
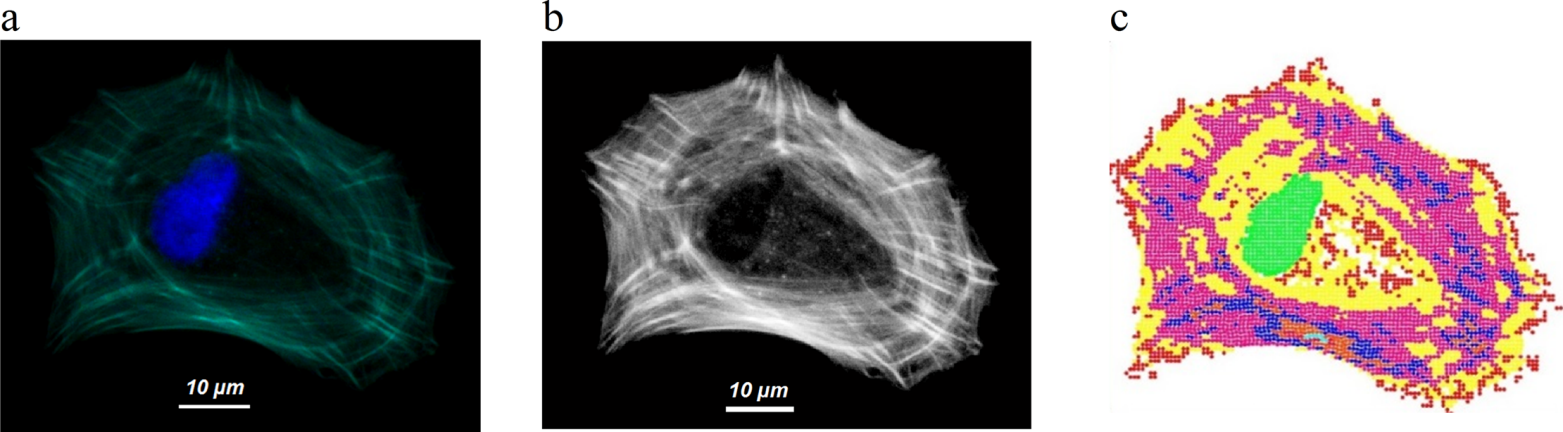
Actin and model images. Cell Example: a) superposition of actin in green and nucleus (DAPI) in blue b) Image of actin network after refinement treatment. c) Pixels were transformed into labelled nodes using a custom built Matlab^TM^ script. Nodes mechanically interacted between each other according to their respective labels.

Actin expression of our Cell Example is shown in Fig 1. To constitute the contractile system representing the actin network, pixels of the image were transformed into interacting nodes labelled according to actin density. Interactions between nodes acted as pre-strained elastic rubber bands whose stiffness was proportional to local actin density (Fig 3). Yet at this point, the coefficient of proportionality, **a** of each cell, remained unknown.

### Solving for “a”, the missing link between actin density and stiffness

As the coefficient **a** increased, so did tensions within the model and forces exerted on focal adhesion nodes. To find **a**, we did a parametric analysis for each cell to compared the total force generated by the model on focal adhesions with the one estimated experimentally in correlation with focal adhesion size. When both numerical and experimental adhesion forces were equivalent, the value of ***a*** was considered to have been found.

This parametric analysis enabled us to evaluate the coherence of the model and potential variations. Internal compression bearing elements such as microtubules were often neglected in previous literature; we thus decided to test two scenarios where internal compression forces were either possible (MT+) or not (MT-).

**Fig 4:**
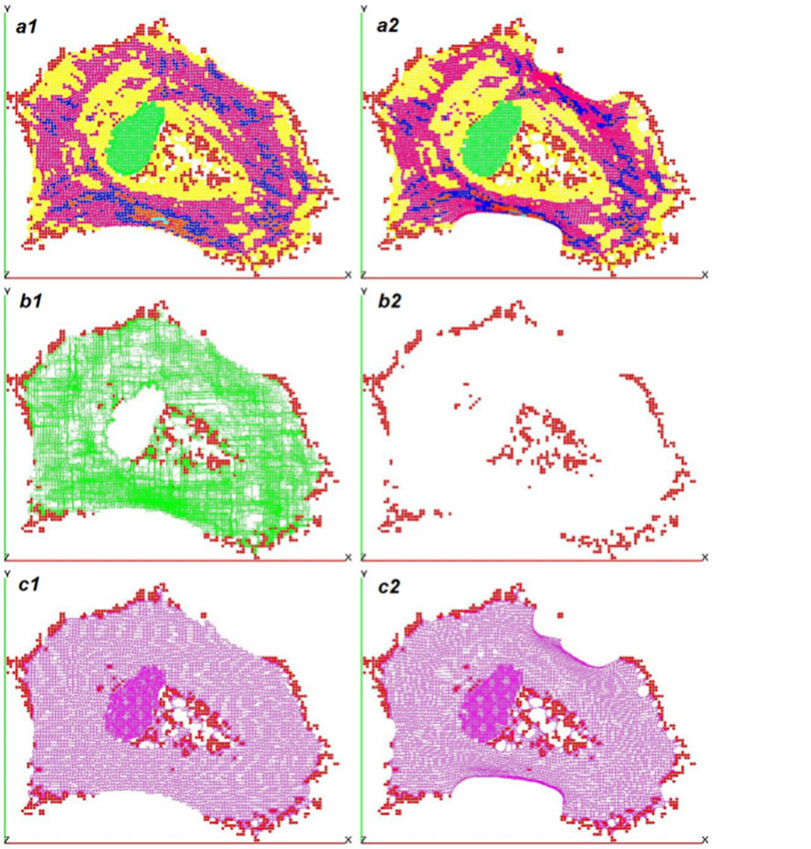
Model images. Cell Example: Left: scenario with microtubules (MT+); right scenario without microtubules (MT-). A) Interaction nodes and spherical contactors, network of B) compressions, C) tensions. Model MT+ was able to retain its initial shape contrary to model MT-. This seems to indicate that the intracellular compression network contributes to cell morphology.

- Scenario MT+: internal compression force enabled (with microtubules) (Fig 4A1, 4B1 and 4C1)
- Scenario MT-: internal compression force disabled (without microtubules) (Fig 4A2, 4B2 and 4C2)

We applied the parametric analysis for **a** to both scenarios. Fig 4 shows the shape of the modeled Cell Example after 600 simulation steps and the distribution of internal forces for scenario MT+ & scenario MT- (for a = 4).

**Fig 5:**
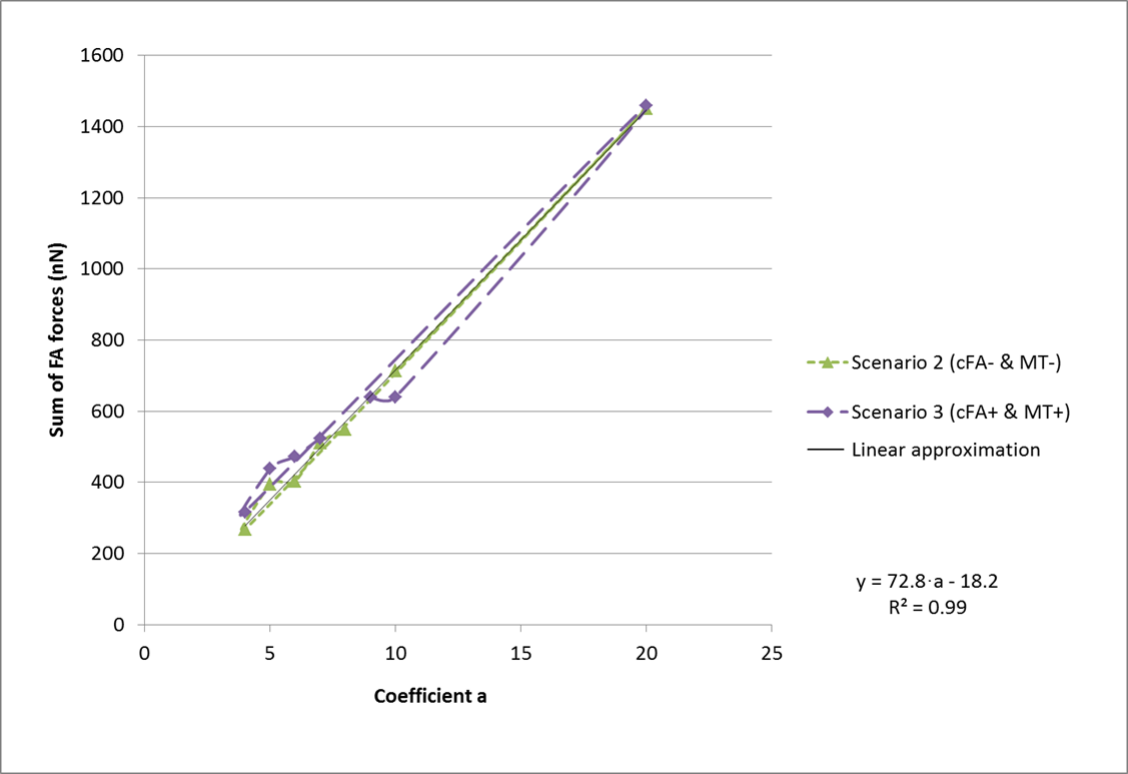
Sum of FA forces. For each simulation of scenario MT^+^ we made coefficient **a** vary between 1 to 15, the sum of focal adhesion forces outputted by the model ranged from F_a = 1_ to F_a = 15_, which included the value. Knowing that the sum of focal adhesion force magnitudes was F_measured_, we used the linear interpolation from Scenario MT^+^ (Eq 10) to solve for **a**.

After several calculation steps we noticed that the cell models with intracellular compression forces enabled would pull less than if intracellular forces were disabled. On some cells we noticed that intracellular compression was necessary to maintain cellular shape. For both scenarios, the sum of focal adhesion forces generated by the model was considered almost linearly proportional to the value of **a** (Fig 5). Such that:

$$\begin{array}{cc} \begin{array}{ccc} F_{measured}=\alpha \cdot \text{a}-\beta & with & \end{array}\{\begin{array}{c} F_{measured}\;:sum\;of\;focal\;force\;magnitudes\\ \alpha :slope\;coefficient\\ \beta :constant \end{array} & \end{array}$$ (9)

On the modeled cell shown in previous figures we found determination coefficients R^2^ close to 1. The following linear interpolations link the sum of FA forces (**y**) to coefficient **a**:

$$\begin{array}{cc} \begin{array}{cccc} Scenario\;MT^{+}: & y=72.8\cdot \text{a}-18.2 & ; & R²=0.99 \end{array} & \end{array}$$ (10)

$$\begin{array}{cc} \begin{array}{cccc} Scenario\;MT^{-}: & y=77.0\cdot \text{a}-19.9 & ; & R²=0.95 \end{array} & \end{array}$$ (11)

The values of **R**^**2**^, **y** and **a** varied quite significantly for each modeled cell. It was none the less used to determine the value of a (N.B. in all cases increasing time step resolution would bring R^2^ closer to 1 and β closer to 0).

### Stability of the model and computation quality

The free run length is an indicator of divided medium stability. It represents the average displacement per time step. Thus, a low value would indicated a stable structure, while a high value indicated an unstable structure. The overall model stability at the end of computation is compared for both scenarios the free run length was lower when microtubules where enabled (MT+) then when they weren’t (MT-).

We found that the free run length was almost linearly proportional to **a**. The structure became less stable as overall rigidity increased for both scenarios (MT+ & MT-). The scenario allowing generation of compression force had significantly lower free run length values than its purely tensile counterpart, which implies that compression forces contribute to the system’s stabilization.

In this simulation, the node masses were not an important parameter, since local acceleration remained negligible with respect to externally applied forces. We nonetheless set node masses to represent an equivalent density close to that of water.

### How do forces equilibrate the cell structure?

Unsurprisingly the total amount of tensile forces of interactions increases linearly with **a**. On the other hand, the sum of compressive forces generated by sphere/sphere interactions is not linearly proportional to “**a**”. In some cells most of the mechanical load that counteracted actin filament tension transited through the substrate instead of the compression bearing elements of the model, in other cells intracellular compression bearing elements were necessary to stabilize the cellular structure.

In the model, compression interactions were about half as numerous compared to tensile interactions. In fact, the nodes of the model were involved in a tensile equilibrium that maintained them spread out, limiting the number of compressive contacts between neighbor nodes.

The number of tension interactions varies between scenarios, but remains the same independently of the slope to rigidity gradient. This implies that a variation of rigidity does not drastically influence the structural configuration of the model.

**Fig 6:**
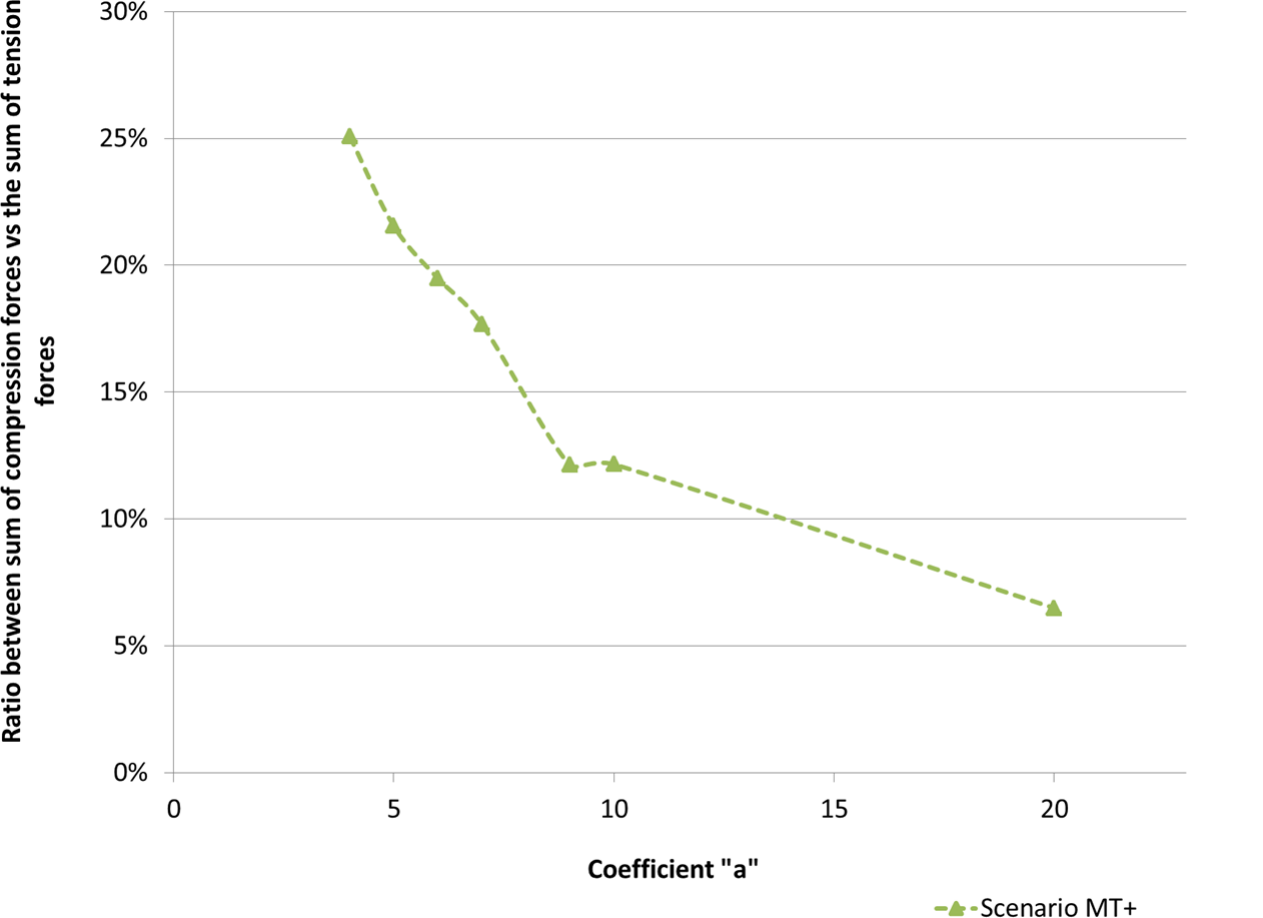
Ratio between the sum of compression forces vs the sum of tension forces. Cell Example: ratio between the sum of all inter-compression magnitudes over the sum of all inter-tension magnitudes decreases, as “a” increases. This implies that an increase of tension does not significantly increase compression.

Furthermore, the ratio between the sum of compression forces over the sum of tension forces (Fig 6) clearly indicates that internal tension is predominant over internal compression. An increase of **a** reduces this ratio (Fig 6). Similarly, in the case of scenario MT+, as **a** increased we noticed that the ratio between mean compression over mean tension, varies from 0.3 to 0.07 (Cell Example). In fact, the mean magnitude of inter-compressions remains steady when the mean magnitude of inter-tensions varies significantly. In the mean-time, the number of interactions tensile or compression remained almost constant. This clearly indicates the limited action of internal compression bearing elements and that additional tension is predominantly balanced by the substrate. We therefore conclude that imaging the actin cytoskeleton correctly and determining the intensity of FA forces with precision may be of greater importance than a precise representation of the compression network. For **a** ranging from 4 up to 20 nN, we can notice that mean inter-tension and mean inter-compression forces respectively range between 1.0 and 5.2 nN and between 0.3 and 0.4 nN (Cell Example). It may be worth noticing that the presence of internal compression bearing elements within the structure does not influence actin tension values significantly.

In our Cell Example, scenario MT^+^ for **a** ranging from 4 to 20 nN, the sum of focal adhesion forces outputted by the model ranged from 266 nN to 1458 nN, which includes the values 336 nN we found based on focal adhesion size. Based on MT^+^ results, we obtained a well correlated relation linking mean inter-tension T_mean_ and the sum of focal adhesion force magnitudes “x”:

$${\text{T}}_{\text{mean}}=0.0035\text{x}+0.1594\;(\text{Cell Example})$$

$${\text{R}}^{2}=0.9912$$

Thus by inputting the sum of focal adhesion force magnitudes “x” into the equation we obtained the mean inter-tension value “y”. For a value of 336 nN we obtained mean inter-tension values of 1.3 nN per interaction (Cell Example).

We can also linearly correlate mean inter-tension with **a** in scenario MT^+^:

$${\text{T}}_{\text{mean}}=0.2567\text{a}+0.0764\;(\text{Cell Example})$$

$${\text{R}}^{2}=0.9999$$

We found the same values of T_mean_ with the solved values of “a”:

$$\text{a}=4.4\;\text{nN}=>{\text{T}}_{\text{mean}}=1.3\;\text{nN}$$

However, these results depend the cell and the nodal density. For instance, lowering the number of nodes would artificially decrease the number of interactions and increase the mean tension value per interaction. On the other hand, if the density of nodes increases, the value of mean tension per interaction would decrease. We can thus conclude that nodal density is directly linked to inter-tension magnitude. For this reason, we considered cross-linear tension, i.e. interaction tension divided by the distance between two nodes (d_0_ = 0.58 μm), so that our results could be viewed as being independent from nodal density. When focal adhesion force magnitudes of the model correspond with the ones measured experimentally, we obtain a mean cross-linear tensions of T_mean-cl_ = 2.3 nN/μm (Cell Example).

### Cross-linear tension forces within the cell example

**Fig 7:**
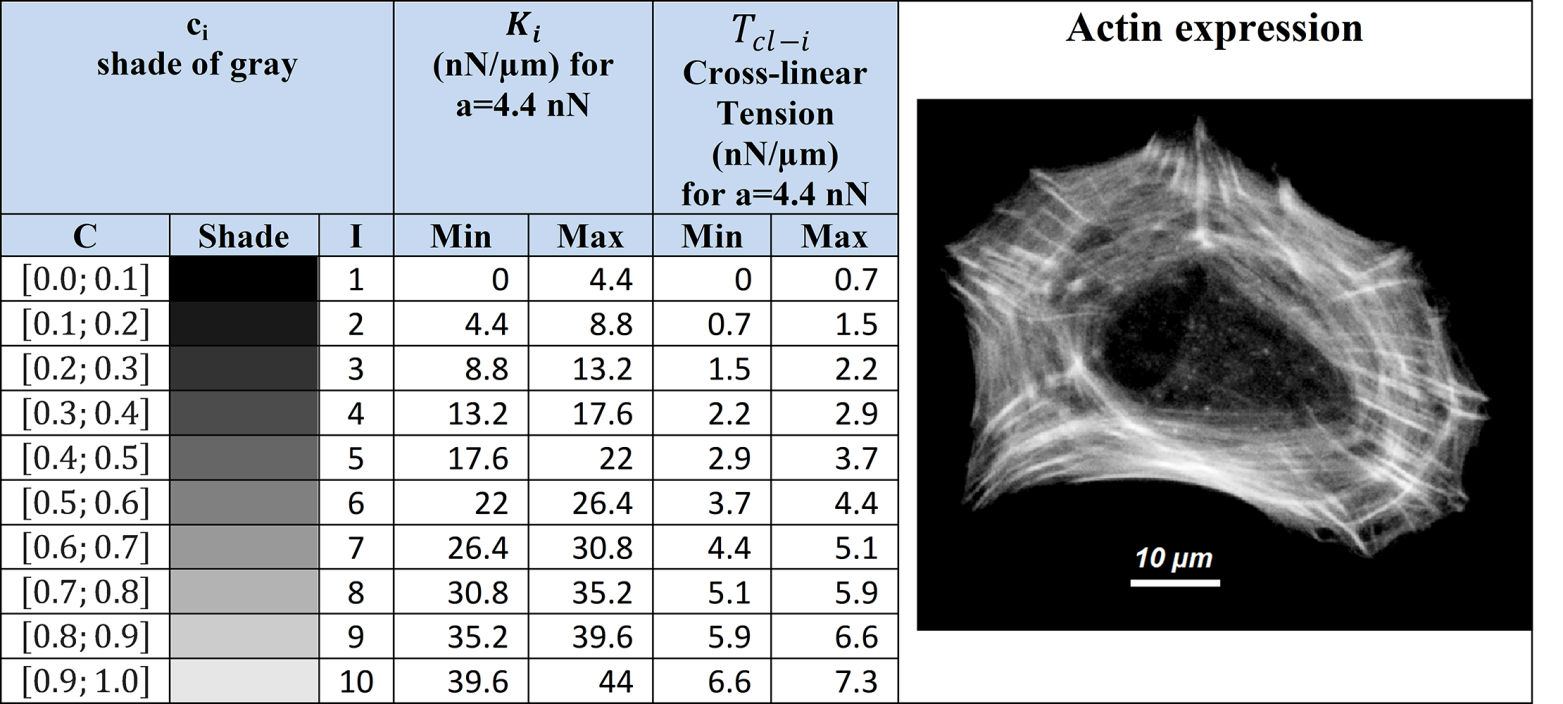
Cross-linear tension forces within the cell. Cell Example: For **a** = 4.4 nN, we have the domain max and min values of ***K*_*i*_** & ***T*_*cl-i*_** in correspondence to gray values, each of which is labeled by a value “***i***”. For each label value “***i***” corresponds an interval of possible cross-linear tensions ***T*_*cl-i*_**, ranging from ***T*_*cl-max-i*_** to ***T*_*cl-min-i*_**. Since the amount of actin detected increase as a pixel gets brighter, i.e. as the label value “i” increases, ***K*_*i*_*& T*_*cl*_** increase as well. When we refer to ***T*_*cl-max*_** we in fact refer to ***T*_*cl-max-10*_**, the highest cross-linear tension value.

The highest cross-linear tension ***T***_***cl-max***_ may reach 7.3 nN/μm while highest cross-linear rigidity may be as high as 44 nN/μm^2^ (Fig 7). Cross-linear tension is not limited to the model, it thus allows direct tension measurement from the actin image. We can thus directly measure the tension crossing a segment i.e. the tension transiting through one or several stress fibers, on the image. All we need is the mean gray value and the width of the fiber or fibers we are considering.

Yet, since the resolution of our model is limited to 0.58 μm and since the number of gray value considered in the model is limited, we turned our attention back to the image which has a greater spatial definition and more precise tones of gray (256 compared to 10).

On the other hand, we recommend that this method should only be applicable to cross sections equal or greater than the definition of the model, meaning in this case anything larger than 0.6 μm. Even if this approach can also interpolate tension trough smaller cross sections, it may under estimate tension values due to diffraction and low resolution, for which both phenomenons tend to average the local gray values.

### Cross-linear tension forces within the cell population

Cross linear tension values within the cell population were calculated using the same solving method as used for the Cell Example. Nonetheless, we noticed that cross-linear tension values shown in Table 1 range from 7.3 nN/μm (Cell Example) to 42.3 nN/μm. On the other hand the cross-linear tension values average 24.1 μm/nN across the eight cells, with a standard deviation of 12.8 μm.

**Table 1:** Cross-linear tension values of the cell population.

*T*_*cl-max*_ (nN/μm)	
Average	24.1
SD dev	12.8
min	7.3
max	42.3

Maximal cross linear tension values within the cell population.

### Tension within the stress fiber of the cell example

**Fig 8:**
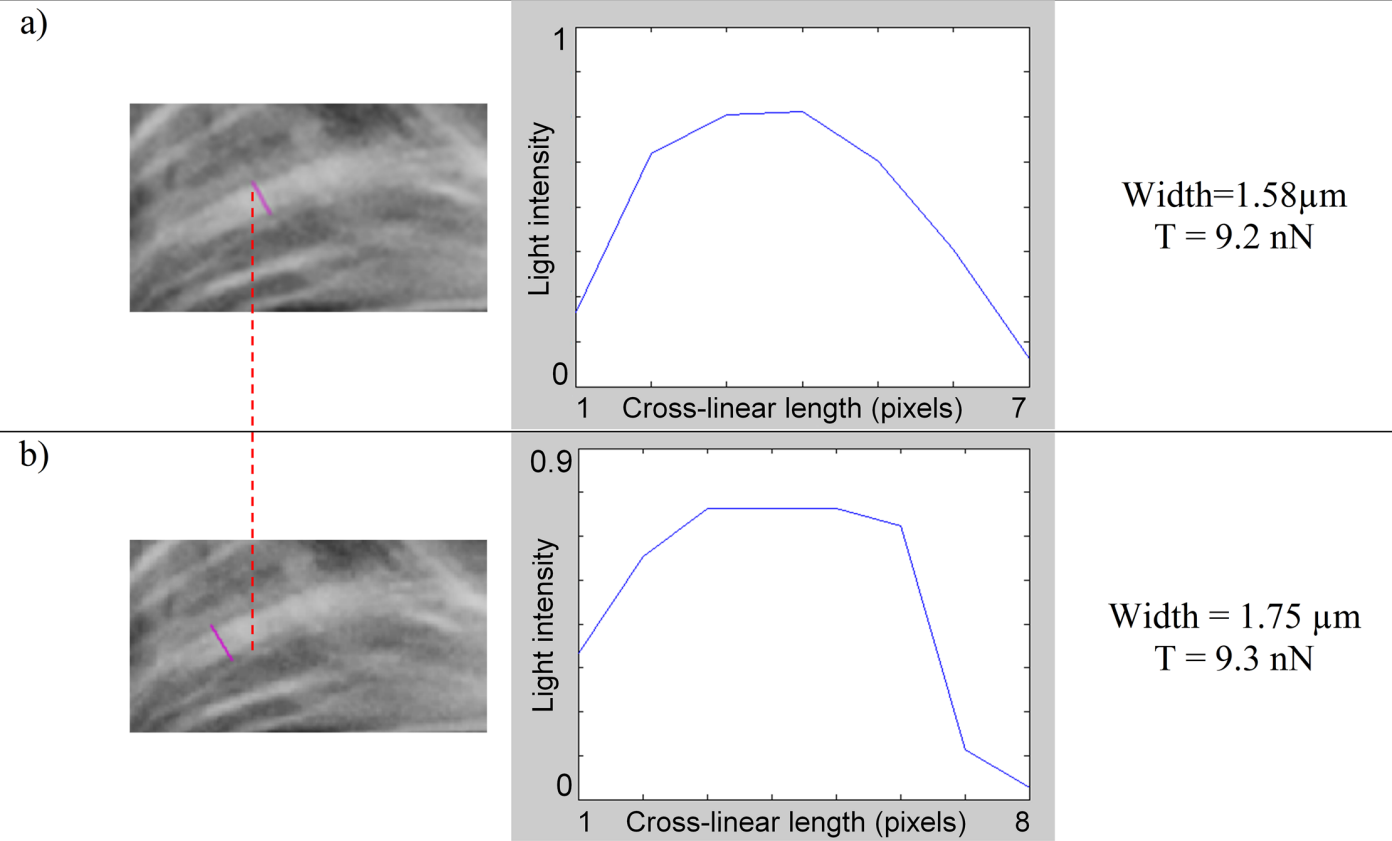
Measuring tension in a large stress fiber at different locations (Cell Example). Measuring tension in a large stress fiber at different locations (a & b). Photos in left column represent a partial view of the actin network. Gray values were measured along the straight pink line running across the considered stress fiber. Pixel gray values measured along each line were plotted on the corresponding graph located in the middle-column (light intensity: 1 = white =“max actin”; 0 = dark = “no actin”). From there the mean gray value was deduced, while the length of the pink line corresponds to the width of the stress fiber (right column). Both cases the width and mean gray values were then used to calculate tension.

**Fig 9:**
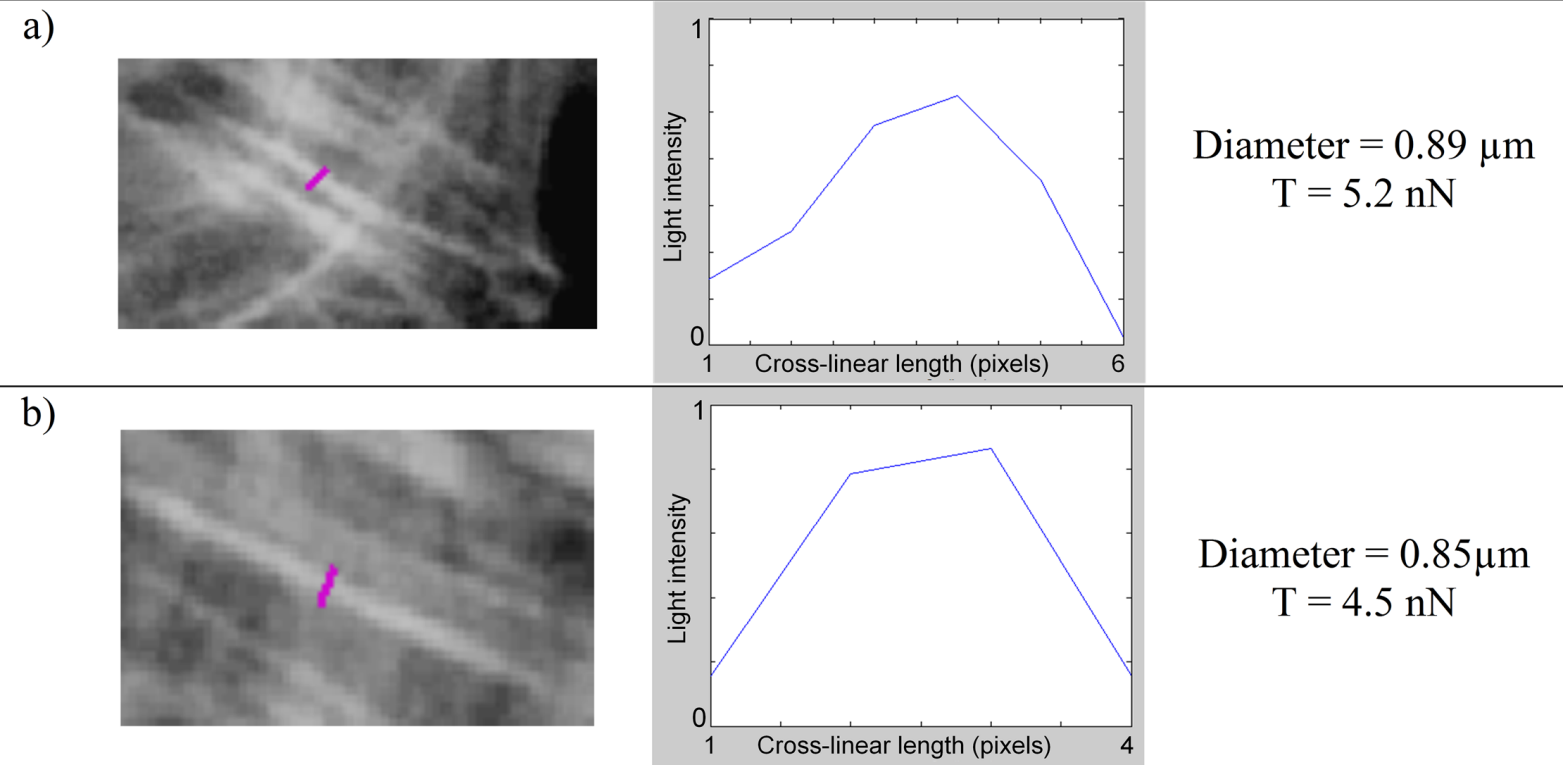
Measuring tension in two different thin stress fibers (Cell Example). See Fig 8 for details.

**Fig 10:**
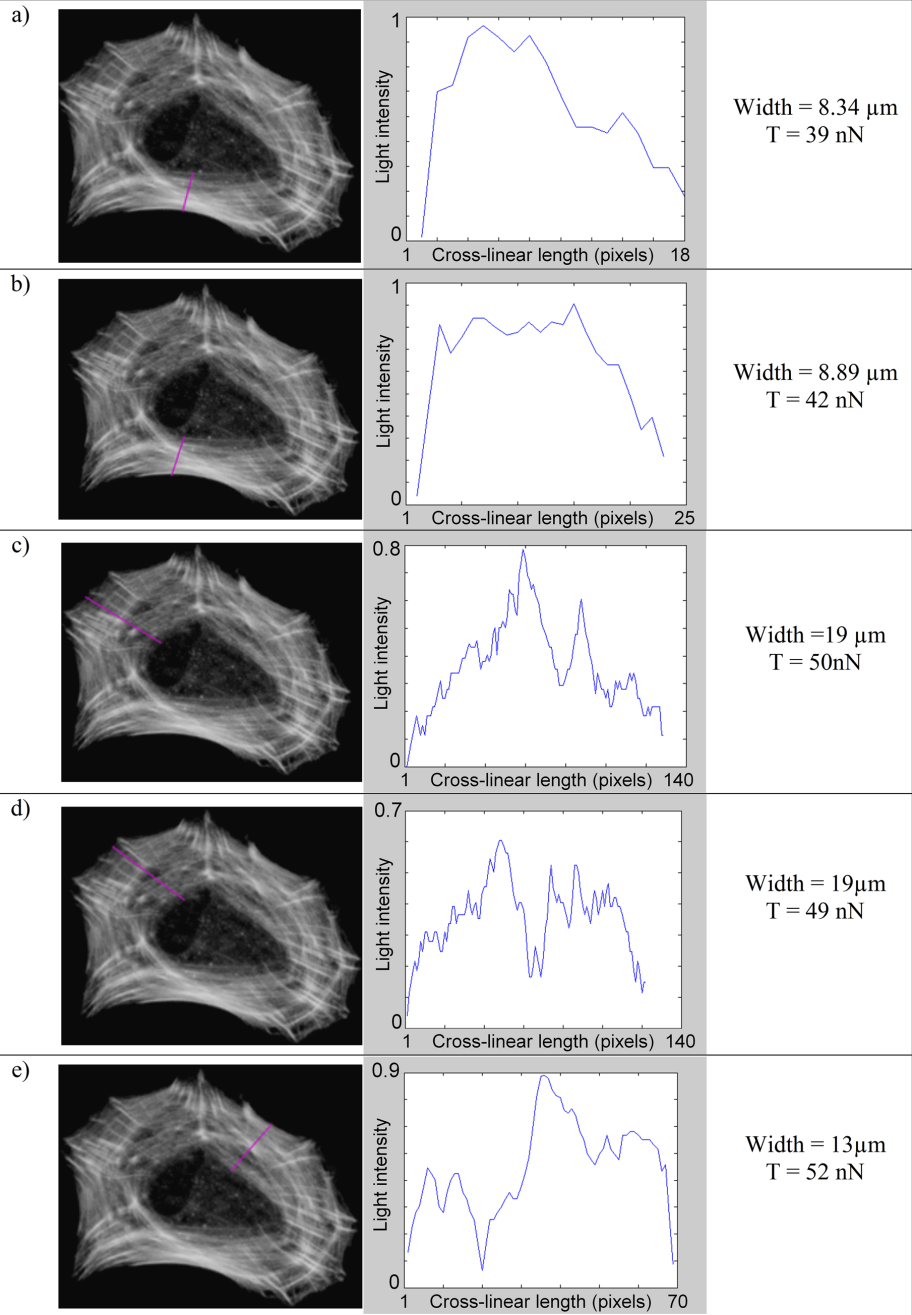
Tension within circular actin network belt (Cell Example). See Fig 8 for details.

**Fig 11:**
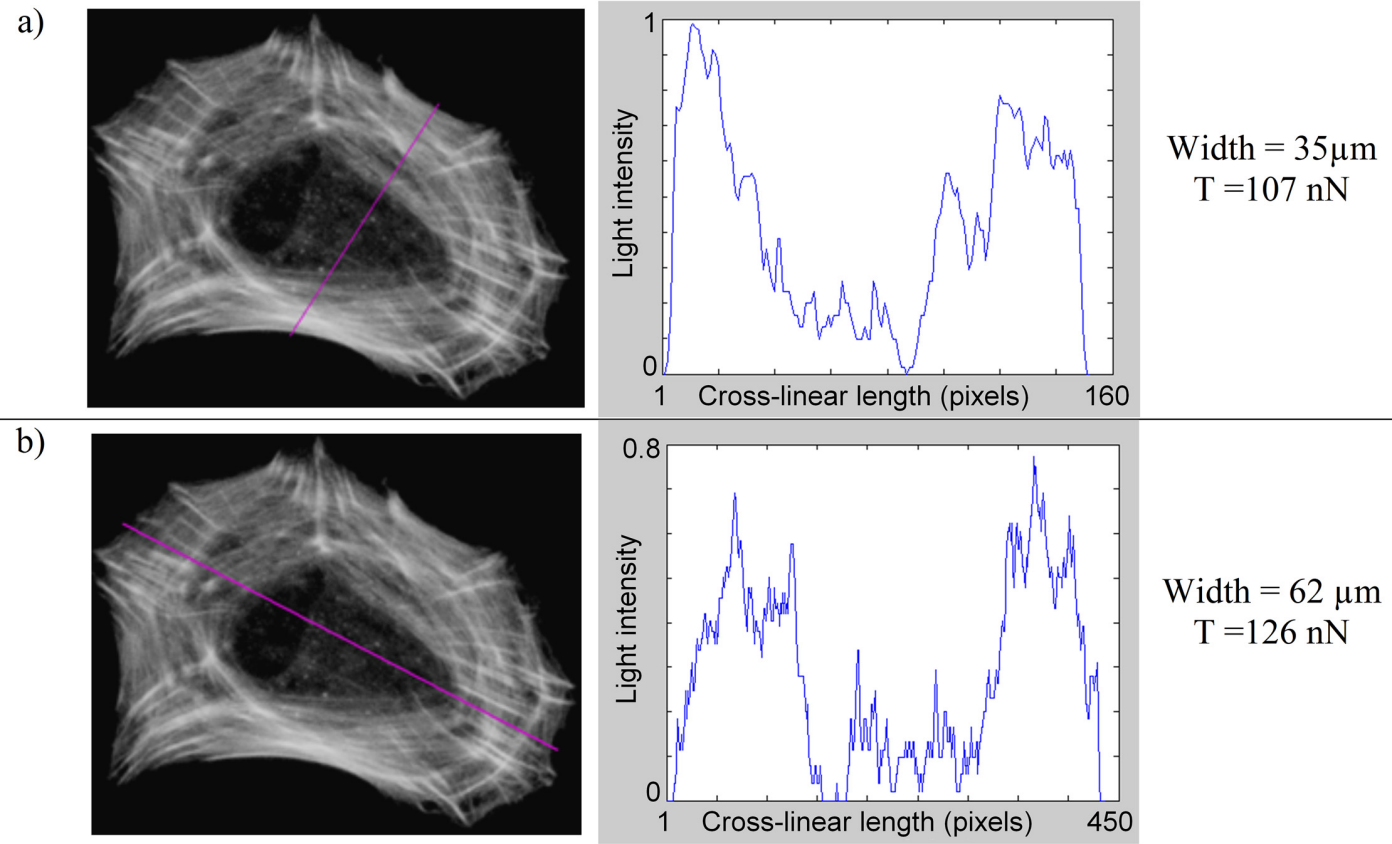
Tension across the whole cell (Cell Example). See Fig 8 for details.

The amount of tension transiting in a stress fiber of the Cell Example was obtained by multiplying the mean gray value ***c***_***mean***_ by the width of the stress fiber and the max cross-linear value (T_cl-max_ = 7.3 nN/μm). Using this approach we estimated intracellular tensions transiting across segments that we drew on the actin image (pink lines on actin images Fig 8, Fig 9, Fig 10 and Fig 11). Pixel values were measured along the segment, while its length indicated the width of the fiber.

In Fig 8A) we thus found T = 9.2 nN of tension transiting through the stress-fiber for a width of 1.58 μm (diffraction is not taken into account when mentioning the width of a stress fiber). We repeated the process for the same stress fiber, at a different location and found a tension value of 9.3 nN for a width of 1.75 μm (Fig 8B). The force value remains very similar even though they have different grayscale histogram profiles. These results indicate that the same tension transit through the stress fiber without being redistributed elsewhere.

Through a thinner stress fiber which we found to be 0.89 μm thick, we obtained 5.2 nN of tension (Fig 9A). Similarly, a tension of 4.5 nN was found for another stress fiber which we measured to be 0.85 μm thick (Fig 9B).

An additional feature of this approach is that it allows the estimation of tension through radial stress fibers (Fig 10). We thus did force measurements within the circular actin network belt (Fig 10). We found equivalent tension magnitudes at different locations ranging from 39 nN to 55nN, with a mean value of 48 nN. These variations can be explained by the way the cytoskeleton belt is structured, because some of the tension might be distributed to other stress fibers linked to focal adhesions. On the other hand, if we look more closely on Fig 10, we notice that cases a & b have very similar results. As could be expected, these are the same group of stress fibers and all additional stress fibers located between the two measurement lines are perpendicular and thus can’t affect tension values significantly. The same principles apply to cases c, d & e. For all three cases we notice that the measurement error always remains below 10%. From these simple tests we can thus conclude that the random measurement error of this method should be below 10%.

To determine the overall intracellular tension, we respectively measured tension across the whole cell, on its short axis (35 μm) (Fig 11A) and long axis (62μm) (Fig 11B), and found 107 nN and 126 nN of tension transiting through each designated section (Fig 11).

This respectively equates to 32% and 38% to the value of the sum of focal adhesion force magnitudes. If we consider this cell to be almost polarized along its long axis, we would thus expect to measure the highest cellular tonus along its long axis, i.e. across the short axis of the cell (Fig 11A). On the contrary, results from Fig 11A and 11B show that the morphological polarization of the cell is not necessarily linked to a polarization of mechanical tensile forces within the cell.

## Discussion

Intracellular traction forces are believed to play a crucial role in mechanotransduction during cellular adhesion process. A better understanding of in vitro observations required the development of mechanical models in the attempt to represent the structural behavior of human adherent cells and propose various mechanical hypothesis [12][13] [14][16]. Although the actin network is the main sub-structural component of the cytoskeleton [9][22], only a few previous models have attempted to reproduce the real organization of the actin stress fibers[16]. To do so, we used a microscope image of the actin network to calculate local tension forces transiting through the cell based on local actin gray values. In the line of works such as Soiné et al. (2015) which reconstructed actin network and computed internal tension, we therein propose a new biofidelic approach whose originality is to take actin density into account. On the contrary to Soiné et al. (2015) who solved tension in stress fibers individually, we computed the contractility of the cytoskeleton by directly resolving the relationship between actin density and local tension. In the end, our aim is for this model to be used to study how mechanical cues may act at a distance within the cell.

### Analysis of the results

#### Actin network, a network of tensions

Independently of the choice of theory between continuous, divided or fluid mediums, it is essential for mechanical cell models to represent force generation and transmission within the actin network which is the key substructure of the cytoskeleton. This means that our actin imaging based model has a significant advantage compared to other published models [12][13][15][23][24][25][26][27].

In our approach, actin amounts were transcribed into tensions within the model. This hypothesis is consistent with experiments which show that stress fiber creation and reinforcement result in an increase in tension. This suggests that the amount of force transiting through a stress fiber could be proportional to its thickness.

#### Modeling actin fibers by pre-stressed elastic interactions

In our model, stress fibers act as purely linear rubber bands with a constant stiffness value and an almost constant strain of 0.2. When we measure tension within stress fibers on actin image, the calculation of force is inherited from the tensile interaction network composing our model. In the following we discuss the consistency of the representation of stress fiber in the model in terms of mechanical and material properties such as the Young’s modulus. For instance, if we consider the stress fiber analyzed on Fig 9B (Cell Example), which possesses a diameter of 0.25 μm, and resulting tension value of 4.5nN combined with a default pre-strain value of 0.2, the equivalent Young’s modulus is equal to 459kPa. Experimental results found in the literature statistically determined a parabolic relationship between stress and strain to characterize the hyperplastic property of stress fibers[10]; using this relationship for a stress fiber of 0.25 μm in diameter and strained by 20%, we found a Young Modulus equal to 322 kPa. We thus find a relative difference of 42% between both results. Furthermore, it is worth noticing that this difference could be encompassed by the standard deviation of the experimental results of the study cited above. Considering the fact that neither the level of strain, nor the diameter of the stress fiber could either be measured with accuracy, we thus consider these results to be very coherent.

When we consider T_cl-max_ which ranges in the cell population from 7.3 to 42.3 nN/μm, we may assume that the minimal value is not so significant since Tcl max would be lower in cells at their initial stage of adhesion. What would be more relevant is to consider the average value (24.1 nN/μm) in relation with the maximal one. There is less than a factor 2. This means that from a cell to another the stiffness of stress fibers may only differ by a factor 2. Furthermore, in the case of a 1μm-diameter stress fiber pulling on a single focal adhesion, it could exert a traction force of 42.3 nN, which is consistent with force magnitude an in vitro cell could exert on a single micro-post [17].

In this study, the mechanical state of the model results from the equilibrium between tensile interactions, only involving slight node displacements. Considering that tensile interactions did not strain further, modeling the hyperplastic properties of stress fibers would not significantly influence the global response of the model. Moreover the static nature of our current work does not require taking viscosity in to account. We can thus conclude that the model is valid to compute intracellular forces within adherent cells. However, hyperelasticity, viscosity and plasticity could be valuable features during dynamic cell deformation or loading, such as oscillatory magnetic twisting cytometry[28].

#### Microtubules play a secondary role to ECM as compression bearing effector

Our results show that a significant increase of inter-tensions did not significantly impact inter-compression within the model. This result is in accord with the experimental results found by other research groups[11][9]. Furthermore the model shows that disabling inter-compression forces increased tug on focal adhesions. This is similar to experimental results found by another group when using chemical agents to depolymerize microtubules[11]. We can therefore conclude that the compression bearing elements of the model counteract actin tension in a similar manner as microtubules would.

As we increased inter-tension magnitudes, the cell model remained stable only involving slight inter-compression variations. In fact, in the model, internal tension is mainly balanced by the ECM compared to microtubules. Experiments have also shown that the ECM is the main counteracting effector to intracellular-tension, especially for cases of elevated intracellular-tension while microtubules tend to play a secondary role as compression bearing elements[11][9]. We can therefore conclude that in a static scenario, the intracellular compression bearing elements used in our model behaved in accordance with previously published experimental results. It thus appears that sphere/sphere interactions have valuable modeling features that include a qualitatively coherent behavior while requiring minimal information input.

#### Tension magnitudes given by the model

The model computed stress fiber tensions ranging between 4.5 and 9.3 nN which is similar to the FA force magnitude measured by Balaban et al. (2001), who measured FA force magnitudes around 10 nN [17]. As the tensile interaction network exerted realistic amounts of force on FA, we can argue that consistent tension magnitudes were generated within the cell model. The model thus yields a coherent relationship between actin density and mechanical force. This allows quantitatively measurements of the amount of tension transiting through the observed stress fibers.

An intracellular tonus index was previously introduced by Milan et al. (2013) as the sum of all stress fiber forces crossing the mid-section of the cell. Based on this definition we found an intracellular tonus of 126 nN which corresponds to 35% of the sum of all FA force magnitudes. In a pre-stressed structure where internal compression is marginal, internal tension cannot be higher than half of overall peripheral forces. It is important to notice that the ratio we found is similar as the one found by Milan et al. (2013).

As mentioned previously, a linear relation exists[17] between the size and force magnitude of FA larger than 1 μm^2^. On the other hand, this linear relationship does not apply to sub-micro squared focal adhesion; this is the reason why we decided to only consider large FA in the present study. However, despite their small size, sub-micrometer squared FA have been reported to support high force magnitudes generally averaging about 15 nN and up to 60 nN [18]. For instance, in the adherent cell we studied here, 40 FAs were in the 0.5 to 1μm^2^ size category and were possibly submitted each to a traction force of 15 nN in average (not published). If we take into account these additional focal adhesions, we can thus conclude that we ought to add another 600 nN to the sum of focal force magnitudes. In this case the sum of all FA forces would be equal to 936 nN, yielding a *T*_*cl-max*_ value equal to 21 nN/μm instead of the 7.3 nN/μm found previously. This difference represents a factor of 2.9 between tension results, which is much more significant than the slight difference observed between scenarios MT+ and MT-. We can thus safely assume that an accurate measurement of FA forces is mechanically more significant than modeling compression bearing agents, such as microtubules.

Improving measurements of FA forces based on FA size, would significantly increase the accuracy of the model results. A potential improvement would be to use of super-resolution microscopy to estimate focal adhesion size, because what appears as a single adhesion in conventional microscopy, can be viewed with a super-resolution microscope as a group of elongated adhesions, between 100 to 280 nm in length. The coefficient of correlation between, focal adhesion size and the pulling forces is significantly higher with super-resolution (R^2^ = 0.711) than with conventional microscopy (R^2^ = 0.459) [19].

On the other hand, the most radical approach to obtain better FA force measurement accuracy would be to use a micro-posts structured substrate to measure force directly due to micro-post deflection[29][18][17]. In the end, even if the results of the model depend on FA forces measurement accuracy, it can nonetheless operate with all of the measurement methods.

### Limits and future perspectives

The representation of tension stress transiting through individual stress fibers would require overcoming the diffraction barrier to better measure their diameter. Super-resolution could be used to fulfill this purpose [30][31][32]. In addition, it would facilitate visual texture recognition to discriminate tension-bearing stress fibers from compression-bearing dendritic actin, which has a woven visual aspect; dendritic network and stress fibers may be represented as different materials with specific mechanical properties. Actin fibers could be modeled as tension interactions between nodes attached to different parts of the dendritic actin network, while dendritic actin could be modeled as a continuous material with compressive stiffness. However, the approach presented herein may not be extended to “mechanically” represent the cytoskeleton down to the molecular interaction scale, for it is bound by the limitations of classical mechanics.

3D acquisition of the cellular structure would allow 3D computation of mechanical stress within the cell[33], and thus help identification of overlaying mechanical pathways which would otherwise appear to be merged when observed in 2D [8]. 3D acquisition would also yield to the estimation of vertical forces exerted on the nucleus[33][34] which influence gene expression[33][35]. As a matter of fact, 3D confocal microscopy allows us to discriminate between ventral and dorsal actin networks. While dual-objective STORM microscopy, allows 3D visualization down to individual microfilaments[30].

On the other hand, in accord with the tensegrity theory, observing the cytoskeletal structure in three dimensions is fundamental to consider the whole mechanical stability and integrity of the cell[8]. Thus using technologies such as dual-objective STORM microscopy combined with the methodology used in our model would be a significant step towards qualitative and quantitative understanding of the mechanical interactions linking the nucleus to the extracellular matrix.

## Conclusions

The scope of this work is to redefine the way we mechanically model the cell. Many macro-scaled biomechanical models are based on medical imaging, such as MRI scans [36][37][38] and CT scans, which are then transformed into numerical models using software such as Mimics®, 3-Matic and Avizo Fire®. This very modeling phenomenon which is currently rising among macro scaled biomechanical models, gives credit to the idea that imaging the cell to generate a mechanical model is a promising research perspective. We thus think that in the future, imaging-based biomechanical modeling will contribute to a more detailed understanding of mechanotransduction within the cell.
